# Profile of Humoral Immunity and B Cell Pool in Infection with the SARS-CoV-2 Prototype Strain and AZD1222 (ChAdOx nCoV-19) Vaccination

**DOI:** 10.3390/vaccines13020101

**Published:** 2025-01-21

**Authors:** Débora Familiar-Macedo, Elzinandes Leal de Azeredo, Elba Regina Sampaio de Lemos, Paulo Vieira Damasco, Luzia Maria de-Oliveira-Pinto

**Affiliations:** 1 Laboratório das Interações Vírus Hospedeiros, Instituto Oswaldo Cruz, Fundação Oswaldo Cruz (IOC/Fiocruz), Rio de Janeiro 21040-360, Brazil; deborafamiliar@gmail.com (D.F.-M.); elzinandes@ioc.fiocruz.br (E.L.d.A.); 2 Laboratório de Hantaviroses e Rickettsioses, Instituto Oswaldo Cruz, Fundação Oswaldo Cruz (IOC/Fiocruz), Rio de Janeiro 21040-360, Brazil; elemos@ioc.fiocruz.br; 3 Rede Casa Hospital Rio Laranjeira e Rio Botafogo, Rio de Janeiro 22240-000, Brazil; paulovieiradamasco@gmail.com; 4 Disciplina de Doenças Infecciosas e Parasitárias, Departamento de Medicina Geral, Universidade Federal do Estado do Rio de Janeiro (UNIRIO), Rio de Janeiro 20270-004, Brazil; 5 Disciplina de Doenças Infecciosas, Departamento de Medicina Interna, Universidade do Estado do Rio de Janeiro (UERJ), Rio de Janeiro 20551-900, Brazil

**Keywords:** COVID-19, AZD1222 (ChAdOx nCoV-19) vaccine, humoral response, B cell subsets

## Abstract

Background/Objectives: Understanding the behavior of B cells during infection and vaccination is important for determining protective humoral immunity. We evaluated the profile of humoral immunity and B cell pool in individuals who were acutely infected with SARS-CoV-2, recovered from COVID-19, or received two doses of the AZD1222 vaccine. Methods: Peripheral blood mononuclear cells (PBMCs) from these individuals were subjected to in vitro stimulation to promote the differentiation of B cells into antibody-secreting cells (ASCs), and the ELISpot evaluated the abundance of pan and SARS-CoV-2 Spike S1-reactive IgG+ ASC. Stimulated PBMCs were characterized using flow cytometry. Culture supernatants were assessed for soluble B-cell-activating factors. The IgA and IgG for the S1 were evaluated through ELISA. Results: The recovered individuals displayed a robust S1 ASC compared to acute and vaccinated individuals. Although the frequency of total B cells or B cell subsets did not vary among the groups, plasmablast cells were increased in naïve and double-negative B cells in the acute, recovered, and vaccinated individuals. Similar IgA and IgG production appeared to be present in the acute and recovered individuals. During vaccination, more IgG is produced than IgA. In acute patients, BAFF levels were positively correlated with total B cells and IgG+ plasmablast cells but negatively correlated with IgA+ plasmablast cells. Conclusions: Vaccination and natural infection with COVID-19 induce a differential profile and functionality of B cells. We suggest that new vaccines against COVID-19 incorporate molecular adjuvants that regulate B lymphocyte functionality and consider the beneficial aspects of the IgA response in addition to IgG.

## 1. Introduction

Protective immunity is expected to occur after a natural infection, especially in those who recover from the disease before vaccination or when individuals are vaccinated against certain pathogens. Immunological memories are generated in all situations. The principle of immunological memory is to help an individual to be protected from disease. CD4+ and CD8+ T cells, B cells, and long-lasting antibody responses are the multiple branches of the adaptive immune system that develop from immunological memory. Substantial advancements have been made in understanding the memory of SARS-CoV-2 infection and COVID-19 vaccines [1]. Despite advances in understanding COVID-19, it is highly dynamic, and new virus variants have frequently emerged. Therefore, vaccine efficiency must be constantly evaluated. Furthermore, there is still a lack of information regarding which essential immunological elements are induced in recovered symptomatic infections or even in vaccinated people who are effectively protected. Since introducing the BNT162b2 mRNA vaccine in late 2020, scientists have been plagued by questions and concerns about efficacy, long-term immunity, vaccine advancement, booster doses, and the risk of reinfection with different variants of SARS-CoV-2. Therefore, more knowledge is necessary to improve vaccine design [2,3,4].

Recently, participants who received one to three doses of inactivated vaccine 1–2 years after infection were compared with those who recovered from a SARS-CoV-2 prototype strain infection without re-infection. The study’s major findings were that antibodies, memory B cells, and T cell immunity against the SARS-CoV-2 prototype were present in recovered patients 2 years after natural infection. In addition, total SARS-CoV-2 T cell responses are able to recognize SARS-CoV-2 variants. An inactivated vaccine improved antibody titers and frequencies of memory B cells but not memory T cells. The study reinforces the need for ongoing surveillance of the duration of infection-induced adaptive immunity and adaptive immunity against viral variants [5].

Newly generated plasma cells either die within days or reside in tissues, such as the bone marrow (BM) or lamina propria of the small intestine, where they can indefinitely persist as high-output antibody-secreting cells [6,7,8]. The induction and maintenance of IgG-secreting cells in the BM have been extensively studied [9], but little is known about IgA-secreting plasma cells in the gut or other tissues [10]. SARS-CoV-2 spike antigen-specific IgG and IgA in patients with COVID-19 mediate viral neutralization. Despite a recent study suggesting that IgA dominates the early neutralizing response to SARS-CoV-2, serum IgA is seven-fold more potent than serum IgG. These data indicate that several types of protective antibodies defend against reinfection and that vaccination regimens should aim for a potent but potentially short-lived IgA response [11].

Different B-cell-activating molecules, such as CD40L, proliferation-inducing ligand A (APRIL), and B-cell-activating factor (BAFF), play important roles in B cell maturation, activation, and differentiation. BAFF is usually expressed in cell membranes, but it can be cleaved, maintaining its functions even in its soluble form. Unlike BAFF, APRIL is intracellularly cleaved and secreted. APRIL is involved in B cell survival, proliferation, antigen presentation, and antibody class switching. Another important member of the TNF family of B cells is CD40 ligand (CD40L). CD40–CD40L interaction on B cells is required to develop germinal centers, induce antibody class switching, and generate B cell memory. CD40L can be cleaved into soluble CD40L (sCD40L), which maintains its ability to bind to the CD40 receptor and exerts biological activity [12,13,14,15].

A related issue is that if the primary immune response to SARS-CoV-2 in the natural infection of patients who develop mild/moderate clinical symptoms is the best-case scenario for an immunoprotective response, the COVID-19 vaccine should induce the same immune response profile. Furthermore, the anamnestic humoral response of the recovered individuals may be the most efficient. Therefore, we hypothesized that patients with mild/moderate COVID-19 and individuals who have recovered from COVID-19 develop a humoral immune response and pool of B cells similar to those vaccinated with AZD1222. These questions are particularly relevant in the search for new vaccines, considering the emergence of many viral variants, limited vaccine supply, and the inequitable distribution of vaccines worldwide [16].

In this study, we evaluated individuals infected with SARS-CoV-2, those who recovered from COVID-19, and those who received two doses of the AZD1222 vaccine. Despite extensive knowledge of COVID-19 and vaccination, few studies have compared the humoral immune response to acute infection in recovered and vaccinated individuals. Our study’s key contribution lies in comparing the humoral immunity profile in natural infection and vaccination during the early phase of the COVID-19 pandemic, before the emergence of variants. This unique perspective allows us to highlight the humoral immunity profile of recovered individuals as a potential model for future, more effective vaccine formulations, thereby paving the way for improved vaccine development.

## 2. Materials and Methods

### 2.1. Study Design and Blood Sample Collection

A cross-sectional study was conducted in patients infected with SARS-CoV-2 and healthy individuals vaccinated with AZD1222 (ChAdOx nCoV-19; AstraZeneca, United Kingdom and Sweden) (Table 1). Two groups of patients with COVID-19 were chosen: those with COVID-19 treated at Hospital Rede-Casa, Rio de Janeiro, Brazil, between March and June 2020 [17], and the recovered group of those who no longer presented with clinical symptoms [18]. Eleven recipients received the ChAdOx1 nCoV-19 vaccine (AZD1222). Plasma was analyzed the day before vaccination (d0) or 14 and 35 days after the booster dose (T1 and T2). The inclusion criteria were adults (male and female) diagnosed with SARS-CoV-2 infection or vaccinated with two doses of AZD1222 following the national guidelines. The exclusion criteria included pregnancy, breastfeeding, teenager and child ages, and vaccination with another vaccine platform. Four of the eleven recipients (36%) reported a previous SARS-CoV-2 infection before vaccination (2020 or 2021). None of the patients reported SARS-CoV-2 infection between the two doses in 2021. Nine of the eleven (82%) recipients reported an episode of SARS-CoV-2 infection in 2022 after two doses. Notably, all vaccines caused asymptomatic infections or mild illnesses, even before or after both doses [19]. Plasma from healthy non-COVID-19 donors was obtained from individuals with no history of infection or clinical symptoms three months before blood collection, which occurred in 2018 and 2019, years before the pandemic.

### 2.2. PBMC and Plasma Isolation

Approximately 20 mL of peripheral blood was collected in BD Vacutainer tubes containing acid citrate dextrose (Becton, Dickinson and Company, Franklin Lakes, NJ, USA). PBMCs and plasma were isolated using Ficoll-PaquePLUS density gradient centrifugation (GE HealthCare, Chicago, IL, USA) and frozen in fetal bovine serum (FBS, Thermo Fisher Scientific, Waltham, MA, USA) containing 10% (*v*/*v*) dimethyl sulfoxide (Sigma-Aldrich, Burlington, MA, USA). The cells were thawed on the day of the experiment and used directly for in vitro assays.

### 2.3. IgG ELISPOT Assay

The Human IgG ELISpotBASIC kit (Mabtech, Sweden) was used according to the manufacturer’s instructions. PBMCs were pre-stimulated in a 96-well culture plate with a mixture of 1 µg/mL of R848 and 10 ng/mL of recombinant human IL-2 (both provided by the ELISpot Kit, Mabtech, Sweden) in RPMI 1640 medium (Thermo Fisher Scientific, Waltham MA, USA) supplemented with 10% heat-inactivated FBS, 1% glutamine, and 1% HEPES (Thermo Fisher Scientific, Waltham, MA, USA) for 48 h at 5% CO_2_. The stimulated cells were washed with RPMI-1640 medium before plating. Sterile ELISpot plates (96-well Filtration Plate Multiscreen^®^ HTS, Millipore, Burlington, MA, USA) were coated with anti-human IgG capture antibody (provided by the ELISpot Kit, Mabtech) or SARS-CoV-2 Spike 1 antigen (1 μg/mL, Sino Biological, Beijing, China) overnight at 4 °C. The number of total IgG+-secreting and S1-specific B cells was determined in wells coated with anti-human IgG or S1, respectively. The plates were washed with sterile PBS and blocked with RPMI-1640 medium/10% FBS for 2 h at room temperature (25 °C to 27 °C). Pre-activated cells (5 × 10^4^ or 5 × 10^5^) were added to IgG- or S1-coated wells in triplicates, respectively, and incubated at 37 °C, 5% CO_2_ for 20 h. After the incubation period, the PBMCs were collected and used for extracellular staining using flow cytometry [20,21,22]. After washing the plates, 1 µg/mL of biotinylated anti-IgG antibody (provided by ELISpot kit, Mabtech) was added, followed by incubation for 2 h at room temperature, and then 0.1% streptavidin–alkaline phosphatase substrate (supplied by the ELISpot kit, Mabtech) was added, followed by incubation for 1 h at room temperature. The plates were washed, and 5-bromo-4-chloro-3-indolyl-phosphate/nitro blue tetrazolium chloride alkaline phosphatase substrate (BCIP-NBT, Sigma-Aldrich, St. Louis, MA, USA) was added. The spots were counted using an ImmunoSpot1S6UV Ultra (Cleveland, OH, USA). The number of antigen-specific IgG-producing cells is expressed as ASC relative to 10^6^ PBMCs. The respondents were those with spots greater than the mean number plus 2× standard deviation of spots from healthy donors, which was greater than or equal to 10.

### 2.4. B Cell Profile Assessment with Extracellular Staining Using Flow Cytometry

The PMBCs recovered using the ELISpot assay were used. Initially, a blocking solution was added to PBMCs for 30 min at 4 °C. After incubation, the cells were washed, and a mix of monoclonal antibodies for surface markers was added (anti-CD16, anti-CD3, anti-CD56, anti-CD14 all FITC, anti-IgA-PE [Miltenyi Biotec, Gladbach, Germany], anti-IgM-APC, anti-IgD-PECy7, anti-IgG-APCH7, anti-CD27-PerCP CY5.5, anti-CD19-V500, and anti-CD38-PE-CF594 [Becton, Dickinson and Company, Franklin Lakes, NJ, USA]) and the LIVE/DEAD™ Fixable Violet Dead Cell Stain Kit (Thermo Fisher Scientific, Waltham MA, USA). After 30 min, the cells were washed, fixed with 4% paraformaldehyde (Millipore Sigma, St. Louis, MA, USA) for 15 min, washed again, and resuspended in PBS. Data were acquired from a Cytoflex S flow cytometer (Beckman Coulter, Brea, CA, USA) and analyzed using FlowJo 10 software (Tree Star^®^, Oakland, CA, USA).

### 2.5. BAFF, APRIL, and sCD40L Quantification

Supernatants from PBMC cultures were recovered using an ELISPOT assay. A LEGENDplex Human B Cell Activator Panel 3-plex kit (BioLegend, San Diego, CA, USA) was used to detect soluble BAFF, APRIL, and CD40L. Undiluted culture supernatants were placed in a 96-well assay plate and mixed with beads for 2 h at room temperature on a shaker (at approximately 500 rpm). Antibodies were added to the wells and incubated for 1 h on a shaker. Streptavidin-PE solution was added to each well. After 30 min of incubation on the shaker, the beads were washed and resuspended in wash buffer. Samples were acquired on a CytoFLEX flow cytometer (Beckman Coulter, Brea, CA, USA), and initial data analysis was performed using the Legendplex software Version 2024-06-15 (BioLegend).

### 2.6. SARS-CoV-2 Serology

According to the manufacturer’s instructions, plasma samples were tested using ELISA to detect IgG and IgA antibodies specifically binding to the SARS-CoV-2 Spike 1 protein (Euroimmun, Waltham, MA, USA). Optical density was measured using a spectrophotometer (Biochrom EZ Read 400, Holliston, MA, USA). Semi-quantitative results were also obtained.

### 2.7. Statistical Analysis

The Kruskal–Wallis test, followed by Dunn’s multiple comparison test, was used for group comparisons. The paired Wilcoxon test was used when data followed a normal distribution. The Mann–Whitney U test was used to compare the frequency between the two groups. The response to kinetic vaccination was analyzed using the Friedman test, followed by Dunn’s multiple comparison test. Differences in variables among groups were considered significant at *p* < 0.05. GraphPad PRISM version 9.01 (GraphPad Software, San Diego, CA, USA) was used for the analysis.

## 3. Results

### 3.1. Recovered Individuals Exhibit a Robust Spike-1-Specific ASC Response

**Figure 1:**
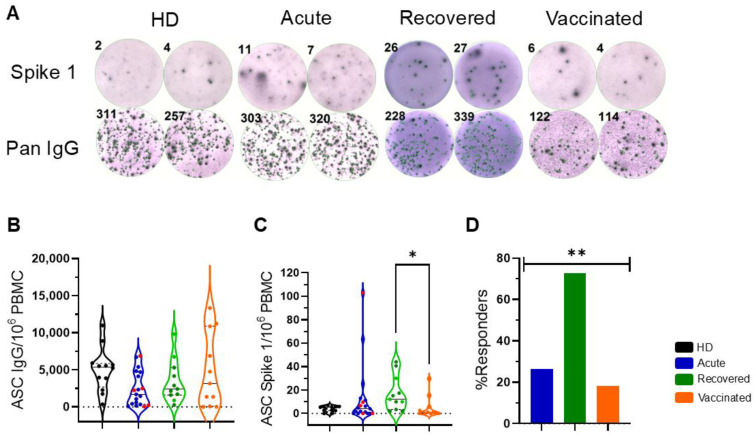
Spike-1-specific antibody-secreting cells (ASCs) in patients with COVID-19 and vaccinated individuals. (**A**) Representative colored spots of pan IgG+ and Spike-1-specific ASCs were demonstrated in healthy donors (HDs), patients with acute COVID-19 (acute), recovered individuals (recovered), and vaccinated individuals (vaccinated). Numbers represent thecounted spots in each well HDs (n = 11) are shown in black, patients with acute COVID-19 (n = 19) are shown in blue, unvaccinated, recovered individuals (n = 11) are shown in green, and vaccinated individuals (n = 11) after 35 days of the second dose of AZD1222 are marked in orange. (**B**) The frequencies of “pan” IgG+ ASCs and (**C**) of Spike-1-specific ASCs relative to 10^6^ peripheral blood mononuclear cells (PBMCs) are demonstrated. Red dots indicate five acute fatal cases. Violin plots show the median (middle line) and describe numerical data distributions using density curves. In (**D**), the frequency of responders, considering people with detected Spike 1 antigen-specific ASCs above or equal to 10, is represented in bars. *p* values were calculated using the Kruskal–Wallis test, Dunn’s multiple comparisons test (**B**,**C**), and the chi-square test t (**D**). * *p* < 0.05, ** *p* < 0.001.

The ELISpot technique was used to detect the presence and abundance of memory B cells reactive to the SARS-CoV-2 Spike 1 antigen regarding the secretion of IgG+ antibodies (ASCs). As controls, cells from the same individuals were evaluated for their ability to produce “pan” IgG regardless of antigen specificity. The secreted IgG was captured with high affinity in both cases using an anti-IgG capture reagent. Representative colored spots of pan IgG+ and Spike-1-specific ASCs were observed in healthy donors (HDs), naturally infected patients with acute COVID-19 (acute), non-vaccinated individuals recovered from COVID-19 (recovered), and vaccinated individuals after the second dose of AZD1222 (vaccinated) (Figure 1A). B cell ELISpot analysis revealed the ability of cells from all individuals to produce “pan” IgG (Figure 1B). The variable presence and abundance of memory B cells reactive to the SARS-CoV-2 Spike 1 antigen were observed among the acute, recovered, and vaccinated groups, resulting in a difference when the recovered and vaccinated groups were compared (Figure 1C). After the analysis, we calculated the frequency of responders, considering people who had a detected Spike 1 antigen-specific ASC above or equal to 10 (calculated as the mean + 2× standard deviation of HDs). Respondents among the acute, recovered, and vaccinated individuals comprised 26.3%, 72.7%, and 18.2%, respectively. This indicated a statistical difference among the groups, in which recovered individuals constituted the majority of the Spike 1 antigen responders (Figure 1D).

### 3.2. Plasmablast Cells Are Commonly Increased in the Acute, Recovered, and Vaccinated Groups, Although the Frequency of Total B Cells or B Cell Subsets Did Not Vary When Comparing the Four Groups

**Figure 2:**
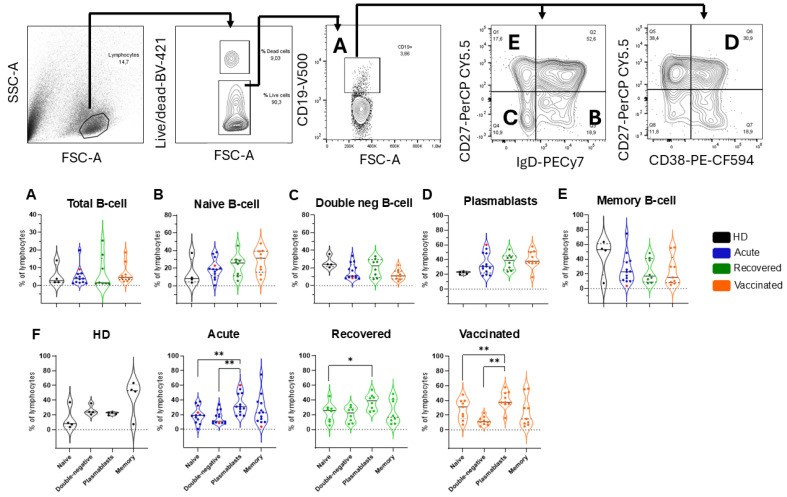
Frequencies of total B cells and B cell subpopulations in all groups of individuals. The gating strategy is displayed by arrows pointing to the next gating level. After selecting the morphological gate (FSC-A x SSC-A), a gate with only viable cells was selected, considering those whose dye reactivity is restricted to cell surface amines, resulting in less intense fluorescence. The frequencies of (**A**) total B cells (CD19+): (**B**) naïve B cells (CD27-IgD+), (**C**) double-negative B cells (CD27-IgD−), (**D**) plasmablast cells (CD27+CD38+), and (**E**) memory B cells (CD27+IgD−). Healthy donors (HD; n = 4) are shown in black, patients with acute COVID-19 (acute; n = 13) are shown in blue, unvaccinated, recovered individuals (recovered; n = 8) are shown in green, and vaccinated individuals (vaccinated; n = 11) after 35 days of the second dose of AZD1222 are marked in orange. Red dots indicate three acute fatal cases. Violin plots show the median (middle line) and describe numerical data distributions using density curves. *p* values were calculated using the Kruskal–Wallis test, Dunn’s multiple comparisons test (**A**–**E**), and the Wilcoxon matched-pairs signed rank test t (**F**). * *p* < 0.05, ** *p* < 0.01. HD, healthy donor.

The frequency of B cells after stimulation with the Spike 1 antigen was based on the classical biomarker, CD19, while previously excluding those expressing CD3, CD16, CD56, and CD14, as shown in the representative analysis strategy of a patient with acute COVID-19 (Figure 2A). The total B cell frequency was similar among the groups (Figure 2A). In addition to the classical biomarkers that define total B cells, CD27, CD38, IgM, and IgD help differentiate the status of B cell subsets, that is, naïve, double-negative, plasmablast cells and memory cells (Figure 2B–E), as shown by a representative dot plot of each B cell subset. No statistical differences were observed when evaluating B cell subsets among the groups (Figure 2B–E). In contrast, the frequency of the subpopulations in each group evaluated individually indicated that in patients with acute COVID-19, an increased frequency of the plasmablast subset was observed compared with that in naïve and double-negative B cells. An increased frequency of the plasmablast subset was observed in recovered B cells compared with that in naïve B cells. Vaccinated individuals showed increased plasmablast cells compared with those in naïve and double-negative B cells, which was similar to the acute patients (Figure 2F).

### 3.3. Soluble BAFF Appears to Regulate Total B Cells in Patients with Acute COVID-19

**Figure 3:**
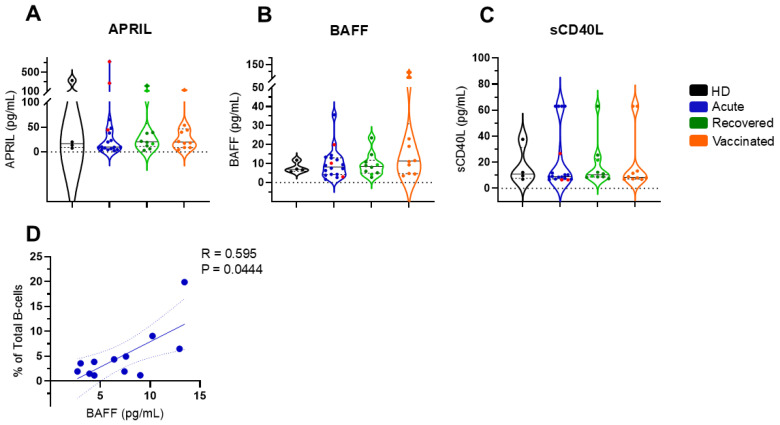
Quantification of soluble APRIL, BAFF, and CD40L in all groups of individuals. For (**A**) APRIL, (**B**) BAFF, and (**C**) CD40L, quantification in healthy donors (HDs; n = 4 in black), patients with acute COVID-19 (acute; n = 18 in blue), unvaccinated, recovered COVID-19 individuals (recovered; n = 10 in green), and vaccinated individuals (vaccinated; n = 11 in orange) after 35 days of the second dose of AZD1222. Five fatal cases have been highlighted in red among the acute cases in blue. Violin plots show the median (middle line) and describe numerical data distributions using density curves. *p* values were calculated using the Kruskal–Wallis test, followed by Dunn’s multiple comparisons test. In (**D**), a statistically significant correlation is demonstrated between the total number of B cells and soluble BAFF levels in patients with acute COVID-19. The Spearman correlation test was used.

After stimulation with the Spike 1 antigen, the PBMC supernatant was recovered, and the B-cell-activating molecules APRIL, BAFF, and CD40L were measured. As shown in Figure 3, no significant differences were observed in these measures between the groups of individuals. Spearman’s correlation statistical test showed that the amount of soluble BAFF was positively correlated with the total B cells in patients with acute COVID-19 (Figure 3B). No other correlations were observed.

### 3.4. Differential Profile of IgA and IgG

**Figure 4:**
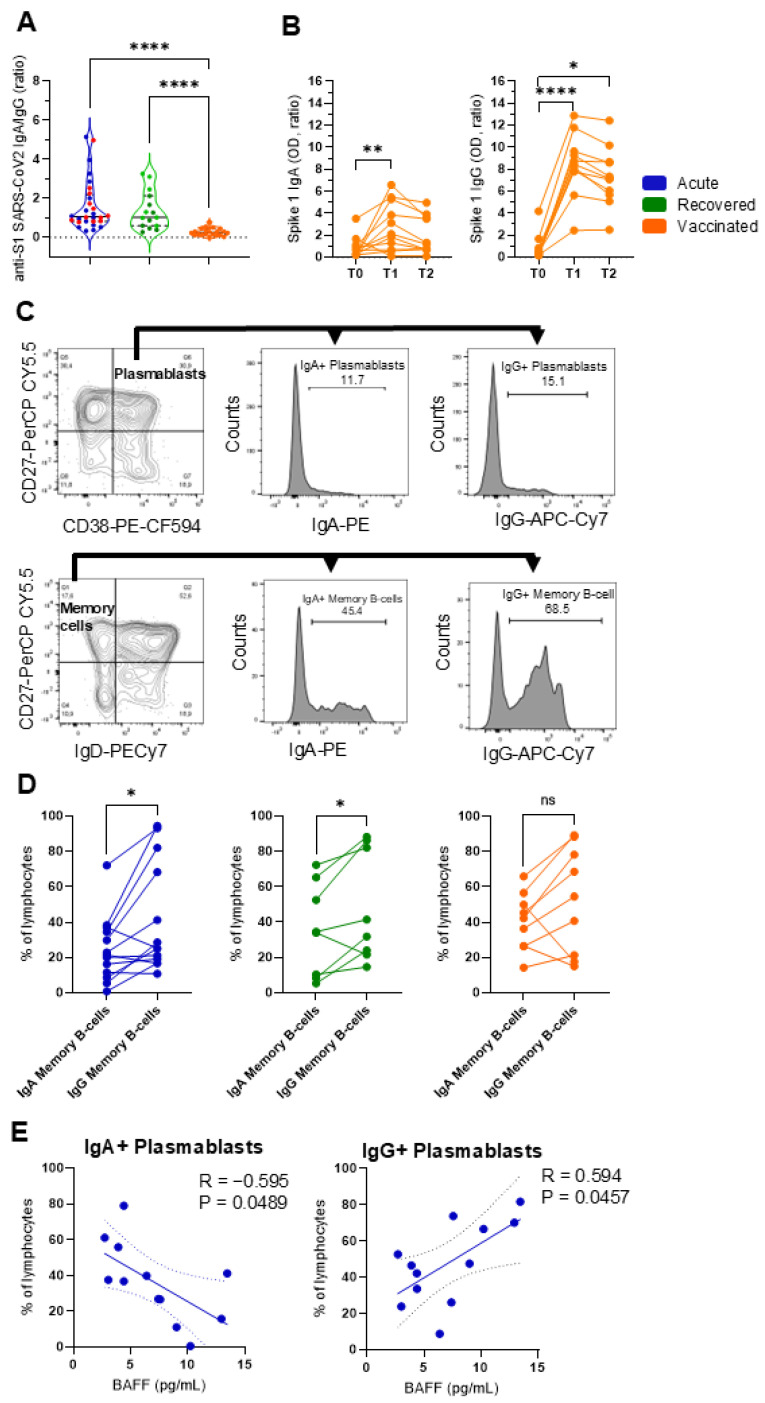
Differential profile of IgA and IgG plasmablast cells and memory B cells. (**A**) The circulating IgA/IgG ratio against the SARS-CoV-2 Spike 1 protein in patients with acute COVID-19 (acute; n = 28 in blue), unvaccinated, recovered individuals (recovered; n = 15 in green), and vaccinated individuals (vaccinated; n = 11 in orange) after the second dose of AZD1222. Red dots indicate eight acute fatal cases. Violin plots show the median (middle line) and describe numerical data distributions using density curves. *p* values were calculated using Kruskal–Wallis and Dunn’s multiple comparison test. Longitudinal (**B**) Spike 1 IgA and Spike 1 IgG are demonstrated before vaccination (T0) and over two doses (T1 = 14 days and T2 = 35 days) in 11 vaccinees. The Friedman test, followed by Dunn’s multiple comparison test, was used. (**C**) The gating strategy is displayed, with arrows pointing to the next gating level. Frequencies of IgA+ or IgG+ are shown in plasmablast cells (CD27+CD38+) and memory B cells (CD27+IgD−) in vaccinated individuals. (**D**) Data with statistical differences from the Wilcoxon matched-pairs signed-rank test in the acute, recovered, and vaccinated groups are presented. In (**E**), a statistically significant correlation is demonstrated between the IgA+ or IgG+ plasmablast cells and soluble BAFF levels in patients with acute COVID-19. The Spearman correlation test was used. * *p* < 0.05, ** *p* < 0.01, **** *p* < 0.0001, ns = non-significant.

Figure 4A shows the ratio of IgA/IgG antibodies directed against the Spike 1 protein of SARS-CoV-2 in all groups. Interestingly, patients with acute and recovered COVID-19 had an IgA/IgG ratio close to 1.0, indicating that the production of IgA and IgG is similar in natural infections. However, these proportions were <0.3 for those vaccinated, suggesting that more IgG than IgA was produced during vaccination. Regarding the kinetics of immunization after the second dose of ChAdOx1 nCoV-19 (AZD1222), it is clear that similarly to IgG, IgA was also induced upon vaccination (Figure 4B).

The inclusion of anti-IgA and anti-IgG antibodies in the analysis allowed us to define subpopulations of IgA− or IgG-secreting plasmablast cells (IgA+ or IgG+/CD27+CD38+) and IgA− or IgG-secreting memory B cells (IgA+ or IgG+/CD27+IgD−) (Figure 4C). This analysis established data from cells recovered after stimulation with the Spike 1 antigen. Patients with acute and recovered COVID-19 but who were not vaccinated showed an increase in IgG+ memory B cells compared to IgA+ memory B cells (Figure 4D). Spearman’s statistical correlation test showed that the amount of soluble BAFF negatively correlated with the frequency of IgA+ plasmablast cells and positively correlated with IgG+ plasmablast cells only in patients with acute COVID-19 (Figure 4E). No other correlations were observed.

## 4. Discussion

This study focused on the role of B cells in COVID-19 outcomes and the development of more effective vaccines. It evaluated patients with COVID-19, recovered individuals, and those vaccinated with ChAdOx1 nCoV-19. Over 70% of the recovered individuals showed a specific ASC response to Spike 1. Plasmablast frequency increased across all groups. IgA and IgG levels were similar in the acute and recovered individuals, whereas vaccinated individuals had higher IgG levels. BAFF levels were positively correlated with total B and IgG+ plasmablast cells in acute patients but negatively correlated with IgA+ plasmablast cells.

Several studies have compared immune responses to COVID-19 vaccines and natural SARS-CoV-2 infection. Wang et al. used single-cell RNA sequencing to analyze immune cells from individuals immunized with the coronavirus vaccine and those with COVID-19. Both vaccination and infection changed the immune cell frequencies, but the effect was stronger during infection. CoronaVac immunization increases HLA class II and IL21R expression in naïve B cells, whereas these markers are downregulated in severe COVID-19 cases [23]. Tartaro et al. found that vaccinated individuals had more antigen-specific memory B cells and polyfunctional CD4+ T cells than recovered patients, indicating differences in adaptive immunity [24].

Humoral immunity is a function of memory B cells and plasma cells, and it is a prerequisite for generating humoral immune memory [25]. Natural infection with SARS-CoV-2 induces a robust population of circulating memory B cells that remain stable for at least eight months, despite the contraction of antibody levels after one month [26]. Byazrova et al. demonstrated that circulating RBD-specific ASC frequencies in patients with COVID-19 ranged from 200 to 4000 ASC per 10^6^ PBMCs. This matches the virus-specific ASC levels observed during other viral infections in order of magnitude and indicates that specific ASCs are generated during the acute phase of COVID-19 [27]. Here, we showed that most recovered individuals displayed a robust Spike-1-specific ASC response (ranging from 2 to 44 ASCs per 10^6^ PBMCs) compared with that in acute COVID-19 (ranging from 1 to 103 ASCs per 10^6^ PBMCs) and vaccinated individuals (ranging from 1 to 30 ASCs per 10^6^ PBMCs). Although detectable, the ASCs obtained in our study were much lower than those of Byazrova et al., which may indicate that the 1 µg/mL antigen coating was suboptimal and that detection only occurred with the highest-affinity ASCs. However, the ASCs comprise <1% of primary and secondary lymphoid organs and blood cellularity, and they can be classified as plasmablast cells, plasma cells, or memory B cells. They represent the final stage of B cell differentiation and are widely distributed throughout the body (secondary lymphoid organs, mucosa, and gastrointestinal tract) [27]. Furthermore, Byazrova et al. confirmed that the acute phase of COVID-19 was characterized by the transient appearance of total and RBD-binding plasma cells [27], indicating that a longitudinal study could better clarify our data. Notably, our ELISpot data showed an increased frequency of antibody-secreting cells (ASC) IgG+ in acute cases after Spike 1 stimulation. Byazrova et al. demonstrated a lack of correlation between the frequency of memory B cells and ASCs, which could explain the low magnitude of our ASCs [27].

Lee et al. studied acute COVID-19 cases and found that B cells in severe cases differentiated into plasma cells more significantly than those in moderate cases. This was associated with increased B cell receptor diversity and higher levels of anti-Spike 1 antibodies. In severe cases, two B cell subsets, atypical memory B cells and an aberrant plasma cell subset, showed inflammatory features, such as elevated cytokine expression and enhanced humoral function, unlike those in moderate cases [28]. These subsets may contribute to disease severity. This study only evaluated mild/moderate, severe, and fatal cases, limiting the ability to categorize patients and analyze their clinical outcomes. More serious cases might have altered the results.

Pušnik et al. found that although Spike-1-specific memory B cells were less frequent in individuals who recovered from mild COVID-19, these cells were functionally superior to those of people with severe disease. Their ability to generate and affinity-mature these cells was linked to IL-21+CD4+ T cells in mild cases and CD40L+CD4+ T cells in severe cases [29]. In this study, most recovered individuals had mild COVID-19, which could explain why over 72% were Spike 1 antigen responders, as indicated by detecting ASCs ≥ 10.

The mRNA vaccines, BNT162b2 (Pfizer-BioNTech, Manhattan, NY, USA and Germany) and mRNA-1273 (Moderna, Cambridge, MA, USA), generated significantly higher levels of IgG and neutralizing antibodies than the adenoviral vector vaccines ChAdOx1 (AstraZeneca, United Kingdom and Sweden) and Ad26.COV2.S (Janssen, Belgium). However, all vaccines trigger spikes and RBD-specific memory B cells, which are essential for long-term protection through rapid immune responses against future infections [30]. In this study, more than 70% of the vaccinated individuals showed an increased frequency of IgG+ memory B cells compared to IgA+ memory B cells after Spike 1 stimulation. However, only 18.2% had ASCs ≥ 10, indicating that immune cell functionality should be assessed using various methodological approaches.

B cell activation, differentiation, and maturation occur in secondary lymphoid organs, which provide signals via cell contact and the secretion of cytokines, chemokines, and soluble factors, such as BAFF, APRIL, and CD40L [31,32]. Studies on BAFF, APRIL, and sCD40L levels in patients with COVID-19 have been inconsistent. Some studies have found increased APRIL and sCD40L levels in recovered patients compared to those with acute COVID-19, whereas others have observed elevated BAFF levels in severe acute cases. Conflicting studies have also shown higher APRIL in severe cases but higher BAFF in mild/moderate cases [33,34,35]. This study found no differences in BAFF, APRIL, or sCD40L levels across the groups. However, in acute patients, BAFF levels were positively correlated with total B cells and IgG+ plasmablast cells and negatively correlated with IgA+ plasmablast cells.

A study focused on developing a DNA vaccine against HIV utilizing the molecular adjuvants BAFF and APRIL to enhance polyreactive B cell maturation and improve the affinity of neutralizing antibodies. Mice immunized with vaccines encoding BAFF or APRIL multitrimers showed germinal center activation, increased secretion of anti-gp120 antibodies, and improved antibody avidity. The authors suggested that the BAFF and APRIL multitrimers are promising adjuvants for vaccines aimed at inducing neutralizing antibodies against HIV-1, making them valuable tools for vaccinology [36].

The ability of the AZD1222 vaccine to induce neutralizing antibody production has been previously demonstrated [37], although this study did not present neutralizing antibody data. A previous study by our group found a positive correlation between IgG antibodies detected using a commercial ELISA kit and neutralizing antibody titers measured using the plaque reduction neutralization test, suggesting that most antibodies detected in patients were neutralizing [17]. Given the benefits of BAFF in enhancing humoral response during infection, incorporating BAFF molecular adjuvants into COVID-19 vaccines could improve protection by fostering a stronger humoral response.

Sterlin et al. found that the initial SARS-CoV-2-specific humoral responses in patients with COVID-19 are predominantly IgA [11]. This is due to the higher production and secretion of IgA in the mucosal tract, which helps control viral spread and lung damage [38]. Although the two doses of AZD1222 vaccine induced both IgA and IgG antibodies, the IgG response was more prominent. In contrast with vaccination, our results show that natural SARS-CoV-2 infection leads to similar levels of IgA and IgG production in acute and recovered individuals. A recent study has suggested that IgA plays a crucial role in the early neutralizing response to SARS-CoV-2, with serum IgA being seven times more effective than serum IgG. Therefore, vaccination strategies should stimulate various antibody types, including IgA, to enhance the effectiveness of vaccines in preventing reinfection [11].

Studies in the literature indicate in most cases that heterologous vaccine regimens with inactivated and adenovirus-vectored vaccines, compared to their counterparts, demonstrate considerable efficiency in increasing the immune responses of vaccinated individuals, paving the way for promising developments of future vaccines or in cases of scarcity of vaccines [39]. Wanlapakorn et al. demonstrated that participants who received the heterologous CoronaVac vaccine followed by AZD1222 had higher levels of anti-RBD IgG with greater neutralizing antibody activity against the original Wuhan variant and all variants of concern than two doses of the homologous CoronaVac or AZD1222 [40]. On the other hand, one study found an increase in systemic reactogenicity following vaccination with heterologous AZD1222-BNT162b2 regimens compared to homologous vaccine regimens [41], highlighting the need for further studies to understand heterologous regimens and engage in ongoing scientific inquiry.

This study had some limitations. The sample sizes of the patients and the vaccinated individuals were small. In addition to male gender, advanced age, pre-existing comorbidities, and racial/ethnic disparities are risk factors related to COVID-19 morbidity [42]. In our current work, we did not observe any statistical difference between the groups regarding gender. However, 87.5% of the recovered patients were female, a highly expected feature of the recovered group, which further validates the credibility of our study. We cannot rule out the possibility that gender constitutes a bias, but this impact can only be assessed in a larger cohort. Furthermore, the four groups are not comparable in age, with older adults being more frequent in the acute group. We need more participants to investigate the impact of age to assess these risk factors. Nevertheless, we aimed to explore the trials to better understand the qualitative and functional profiles of the B cells. Additionally, we only evaluated individuals vaccinated with the second dose at two time points, with one being just 35 days after the second dose. Analyzing those vaccinated after a longer interval would be valuable. Our previous evaluations of vaccinated individuals with natural infection and those without did not reveal significant differences. However, it is important to acknowledge that the small number of vaccinated individuals may have made our analyses more challenging. Furthermore, we focused solely on the ChAdOx1 nCoV-19 (AZD1222) vaccine profile; assessing the B cell profiles of other available vaccines would also be beneficial. Finally, we did not consider the response of B cells to the variants.

## 5. Conclusions

Based on our findings, although the proportion of total IgG-secreting cells per million PBMCs was comparable across the four groups, individuals who recovered from infection produced more antibodies specific to the SARS-CoV-2 S1 protein than vaccinated individuals. Furthermore, the proportion of those producing anti-S1 secretory cells is higher in the recovered group than in the acutely infected or vaccinated group. One recommendation to improve future COVID-19 vaccines is to incorporate molecular adjuvants, such as BAFF, to enhance B cell functionality. Equally important is the need to consider the IgA-producing B cell response, which plays a significant role alongside IgG antibodies in the immune response to COVID-19. This understanding can enlighten us regarding the complexities of the immune response.

## Figures and Tables

**Table 1:** Demographic and clinical characteristics of patients with COVID-19 and vaccine recipients.

	HD	COVID-19		ADZ1222	*p*
		Acute	Recovered	Vaccinated	
Total	11	28	15	11	
Female (%)	64.7	50	87.5	63.6	
Age (years) ^a^	32 (25–61)	55 (24–97)	35 (23–51)	33 (25–61)	HD vs acute * acute vs. vaccinated *
Days after symptom onset ^b^	NA	7 (2–19)	23 (17–38)	NA	
Hospitalization (%)	NA	32	0	NA	
Death (%)	NA	28.6	0	NA	

^a^ Data are expressed as median (minimum–maximum); ^b^ Acute symptoms reported by participants. NA: not applicable. (*) Bold values denote statistical significance (*p* < 0.05). The non-parametric Kruskal–Wallis test was used for quantitative variables (age and number of days after symptom onset). The appropriate chi-square or Fisher’s exact test was used for categorical variables. HD, healthy donor.

## Data Availability

The authors will make available raw data supporting the conclusions of this article without reservation.
